# Physiology

**Published:** 1881-02

**Authors:** 


*Collaborators:*

DR. L. W. CASE, DR. R. TILLEY,
DR. BYRON W. GRIFFIN, DR. W. L. DORLAND,
DR. H. D. VALIN.

### PHYSIOLOGY.

PHYSIOLOGICAL CHEMISTRY.—The Result of Experiments to Diminish the Malignancy of Fowl Cholera. Communicated to the Academy of Medicine of Paris, at its Meeting of October 26th, 1880. By Pasteur.

I take the liberty of recalling the following from among the various results which I obtained in my investigation of the affection commonly known as fowl cholera, and which I had the honor to report to the Academy.

1. Fowl cholera is a malignant disease in the first degree.

2. Its virus is formed of a microscopical parasite which is easily cultivated outside of the body of the animals subject to it. Hence the possibility of obtaining a perfectly pure virus, and the irrefutable demonstration that it is the sole cause of the disease and death.

3. The virus offers various degrees of malignancy. Sometimes the disease is followed by death, and at other times it results in a cure after the provocation of morbid symptoms varying in their intensity.

4. The differences noticed in the malignancy of the virus are not simply the result of observation based on natural phenomena, but the experimenter can repeat them at will.

5. As is the case with all infectious diseases in general, fowl cholera has no relapse, or rather its relapses are in inverse ratio to the severity of the first attacks, and it is always possible to keep the virus so that the most malignant sample will give no result at all.

6. Without any desire to affirm any relation between small-pox and vaccine virus, it is evident that from the foregoing facts, there are conditions of the virus of fowl cholera in which this virus, relatively to the most violent attacks, will act in the same manner as human vaccine does to impart an immunity from small-pox. Vaccine virus causes a mild disease, vaccinia, preservative against a graver disease, small-pox. In the same manner, *the virus of fowl cholera has stages of diminished malignancy which communicate the disease, not death, in such a condition that after the animal has recovered, it is proof against the inoculation of a more malignant virus.* In some respects, however, there is a great difference between the two sets of facts, and it is not useless to remark that, concerning knowledge and principles, the advantage belongs to the study of fowl cholera; for, while we dispute yet the relations of small-pox and vaccinia, we are certain that the modified virus of cholera is a derivative of the most malignant virus of that disease; that we get the second through the first; in a word, that their fundamental nature is the same.

The time has come to give explanations of the capital assertion which is the basis of most of the preceding propositions, to-wit, that there are various states of malignancy in fowl cholera; a strange result, indeed, if we consider that the virus of that affection is a microscopical organism which one can handle in a perfectly pure state, in the same manner that we manipulate beer yeast or the micoderma of vinegar. Yet, if we consider calmly this mysterious notion of degrees in malignancy, we cannot fail soon to recognize that it is probably peculiar to the various species of that group of contagious diseases. Where is, then, the unity in one or the other of the diseases of that group? Citing one instance only, we see epidemics of small-pox of a very malignant character beside others very mild, without the differences being referable to external conditions of climate or to the constitution of the individuals attacked. Do we not see equally epidemics becoming extinct by little and reappearing later and becoming extinct again. Hence the notion of the existence of a varying intensity in the same virus will not at most surprise the physician or the laity, although its scientific demonstration carries with itself a great interest. In the special case before us, the mystery chiefly appears in the fact that the virus being a microscopical parasite, the variations in its malignancy are easily observed. This is what I am going to clearly demonstrate.

Let us take for our starting point the virus of cholera in a very malignant state, in its most malignant state, so to speak. I have explained the manner of obtaining it with this property before today. It consists in taking the virus from a fowl just dead, not from the acute disease, but from the chronic. I have made the observation that the cholera takes at times this last form. Such cases are rare, although it is not very difficult to come across some instances. In these conditions, after the fowl has been very sick, it grows poorer and poorer, and resists death during weeks and months. When it dies, which fact happens after the parasite, leaving other organs, in which it had been confined till then, is taken into the blood where it grows, one can observe that whatever has been the original malignancy of the virus at the time of the inoculation, the sample taken from the blood of the animal, which took so long a time to die, is of a great virulence, which generally kills ten times in ten, twenty times in twenty. This fact being known, let us cultivate successively this virus, in a pure state, in a broth made with the muscle of the fowl, taking every time the seed of one cultivation in the preceding culture, and let us try the malignancy of these various cultures. It is demonstrated through experiments that this malignancy does not change in any sensible manner. In other words, if we agree that two virulences are identical whenever experimenting in the same conditions on an equal number of animals of the same species, the ratio of death being the same in the same time, we will come to the conclusion that the virulence of our successive cultures is the same.

In what I have just said, I have not mentioned the duration of the interval between two successive cultures, or, if we prefer, the duration of the interval from one sowing to the other, and its possible influence on the successive virulences. Let us call attention to this point, however small its importance may appear. In an interval of from one to eight days, the successive virulences have not changed. In an interval of fifteen days the result is the same. In a month's, six weeks', two months', interval, there is no further change observed in the virulences. However, as the interval grows longer, there seem to be certain signs, of little apparent value, that the inoculated virus is becoming weaker. For instance, the rapidity, if not the ratio of death, undergoes delays. In various species of inoculated fowls, we see some very sick ones, lame and drooping, because the parasite in its progression through the muscles, has reached those of the the thigh ; the pericardites are lingering ; abscesses appear around the eyes ; at last the virus has lost, so to speak, a part of its fearful character. Let us once more reach beyond experiments lasting a short time, before beginning a repetition of cultures. Let the duration extend to three, to four, to five, to eight months and more, before studying the malignancy of the development of the new microscopical being. This time the result is thoroughly changed. The differences in the successive malignancies, which, till now, did not appear, or appeared in a doubtful manner, now manifest themselves through prominent effects.

With such intervals between the sowing, it happens that, on repeating the cultures, instead of identical virulences, that is, instead of ten inoculated fowls dying in ten, we meet the ratio of death coming down to nine, eight, seven, six, five, four, three, two, one in ten, and sometimes there is no death at all ; that is, the disease attacks every inoculated individual, but they recover. In other words, in simply changing the mode of cultivation of the parasite, in the sole fact of sowing at longer periods, we have a method to obtain progressively weaker virulences, and finally a true vaccinal virus, which does not kill, but gives a mild form of the disease which imparts immunity from the mortal disease.

One must not believe, for all these alterations, that the results take place with a mathematical regularity and fixedness. Some crops, which have been waiting for five or six months to be sowed, may show a persisting and considerable malignancy, while others of the same origin will already be altered after a lapse of three or four months. We will give soon the explanation of these anomalies, which are apparent only. There is often, indeed, a sort of quick change from an intense virulence to the death of the microscopical parasite in the interval of a short time. While passing from one cultivation to the following, one is surprised to meet an impossibility of reproduction; the parasite is dead. The death of the parasite is otherwise a constant occurrence every time that a renewal of the cultivation has been delayed a sufficient lapse of time.

And now the Academy knows the true motive of the silence which I kept, and the reason why I have asked the liberty of some delay before divulging my method of alteration. Time was an element in my experiments.

But what becomes of the microscopical organism during the course of these experiments? Does it change its form and its aspect, while changing its malignancy in so profound a manner? I could not affirm the non-existence of certain morphological relations between the parasite and the various virulences which it undergoes, but I must own, that, until now, it has been impossible for me to discover them, and that, if they really exist, they are invisible to the eye helped by the microscope, so small would be the virus in that case. The cultivations are alike for all the malignancies. If one seems to perceive feeble changes sometimes, it seems in a short while to be nothing but an accident, and they do not reproduce themselves in the following cultures, something remarkable is that if we take each variety of virulence as the starting point of new cultures, successively repeated at short intervals, the variety of virulence is preserved with its proper intensity. Suppose the case of an altered virus which kills but one time in ten; it retains this virulence under cultivation if the intervals of sowing are not very long. An equally interesting fact, although contained in the general course of the preceding observations, is that an interval sufficient to kill an altered virus spares a more virulent virus which may be thereby altered but which does not necessarily die.

At this point whither we have just come, an important question presents itself; What is the cause of the diminution in the malignancy?

The cultivation of the parasite must necessarily be made in contact with the air, because this virus requires a certain amount of air to live. And it could not be developed without it. Hence it is very natural to ask at once, whether the weakening influence on the virus is not in the contact with the oxygen of the air. Is it not possible that the little organism constituting the virus, remaining in contact with the oxygen of pure air, in the medium in which it has just multiplied, should undergo some modification which would return permanently whenever the organism would be withdrawn from the modifying influence ? We may, it is true, ask ourselves again if some principle of atmospheric air, besides oxygen, some chemical or fluid principle, might not intervene in the accomplishment of the phenomenon, whose incomparable strangeness authorizes all sorts of suppositions.

It is easy to understand that the solution of this problem, in case it should follow from our first hypothesis, that relating to the influence of the oxygen of the air, is easy enough to ascertain, for if the oxygen of the air is the modifying agent of the malignancy, we may very likely find a proof of it in the result of the suppression of oxygen. With this intention, let us make our experiments in the following manner: A convenient quantity of hen's broth being sowed with the most malignant virus, let us fill with it glass tubes to one-third, to three-fourths, etc., of their volume; then let us close these tubes in the flame of a lamp. By the help of the small quantity of air left in the tube, the development of the virus will take place, a circumstance which the eye can perceive in a perturbation of the liquid; the progress of the breed soon absorbs all the oxygen contained in the tube. Then the perturbation ceases, the virus deposits itself on the walls of the tube, and the liquid becomes clear once more. This requires two or three days. The little organism is henceforth protected from the contact of oxygen and will remain in that state as long as the tube remains closed. What will its virulence become this time? To secure a greater certainty in our experiments we shall have a number of similar tubes, and at the same time an equal number of flasks of the same cultivation freely exposed to the contact of pure air. We have mentioned what becomes of the samples exposed to the contact of the air; we know that they undergo a progressive diminution of their virulence; we will not repeat it any more. Let us mention only the sealed tubes protected from the air. Let us open them. Of the one after an interval of a month, and after having reared a crop by sowing a portion of its contents, let us try the malignancy ; of the other also, after an interval of two months, and in this manner a third one, a fourth one, etc., after intervals of three, four, five, six, seven, eight, nine, ten months. This is where I stop. It is remarkable as the experiment proves, that the virulences are always similar to those of the virus which serves in the preparation of the sealed tubes. But for the samples exposed to the air, we find that they are dead by this time, or possessed of the feeblest malignancy.

The question proposed to us is accordingly resolved ; *it is the oxygen of the air which weakens and destroys the malignancy of the virus.* Very likely there is here something more than an isolated fact; we are doubtless in possession of a principle. We have reason to hope that an inhering action of the oxygen of the air, a natural force present everywhere, will show its efficacy on all other viruses. It is, in any case, a circumstance of interest the possible extensive generalization of this method in order to diminish the virulence of diseases, borrowing its virtue from an influence in some sort atmospheric. Have we not a right to presume, from this day, that we must refer to this influence, the limitation of great epidemics in our days as well as in the past? The facts which I have just had the honor to present to the Academy, suggest numerous inductions, proximate or remote. I will believe myself authorized to publish them if I succeed in turning them to demonstrated truths only. H. D. V.

EXPERIMENTS ON THE CERVICAL PORTION OF THE SYMPATHETIC. A Note of Messrs. Dastre and Morat, presented to the Institute, by M. Gosselin.

All that is known of the functions of the sympathetic nervous system is based, almost exclusively, on two experiments of Parfour du Petit (1727), and those of Cl. Bernard and Brown-Séquard (1851). Parfour du Petit demonstrated the ascending direction of the nervous fibers in the cervical portion, a purely anatomical fact. The experiments of Cl. Bernard show the *vaso-constrictor* nerves contained in the cervical sympathetic, whose office is to constrict the blood vessels. The facts which we communicate to the Academy complete these notions and demonstrate the existence of *vaso-dilator* nerves in the same, which antagonize the preceding.

The experiment which establishes this result is that of Cl. Bernard itself, which was a repetition of Parfour du Petit's. Cl. Bernard stated that physiologists before him, as well as himself, for some time, had repeated the famous experiment of Parfour du Petit without discovering its most striking result; it is our turn to add that every physiologist has repeated Cl. Bernard's experiment without discovering its most striking result, at least whenever it is performed on the animal most experimented on, the dog. While all current notions were prevailing against our investigation, the chain of our experiments led us to the discovery of truth.

Here it is. Whenever the cervical sympathetic is irritated, there is produced a primative dilatation, immediate, often enormous, of the blood vessels in the corresponding half of the buccal cavity, that is, in the mucous membrane of the palate, the gums, the lips, and in the integument of the lips and cheeks; in the blood vessels of the upper and lower jaw. The redness becomes intense, and other signs of the dilatation of the vessels appear, as heat, tumefaction, straightening and *umbilication* of the hair. All this is limited to that half of the face corresponding to the irritated nerve. They disappear almost immediately after the irritation has ceased. A definite line separates the scarlet-red region from the pale side, and what makes the phenomena still more remarkable and significant is, the paleness of other organs of the same side, the ear and half the tongue, while those alluded to above are red and congested, in such a way that the contrast in the color of the two sides of the tongue is the reverse of the contrast between the colors of the two sides of the buccal cavity and renders it more striking. These phenomena constantly recurred in our experiments, and are so evident as to be worthy of demonstration before a class, under favorable conditions, that is, provided the mouth be slightly pigmented, the nerve in a condition of rest, and the animal be quiet, or rendered motionless by a small dose of curare.

Were these experiments less evident, their result would be called paradoxical, since they are exactly opposed to the current notions based on the experiment of Cl. Bernard and Brown-Séquard. But, we hasten to say, that it does not contradict that famous experiment more than the latter did that of Pourfour du Petit; they simply complete it. The researches which we pursued since four years, regarding the innervation of blood vessels, led us to the discovery of the first cutaneous vaso-dilator ever announced, that of the ear; and we found it in the sympathetic. In the same manner, we found in the sympathetic the dilators of the lower extremity, of the upper extremity and various viscera; finally, the origins of the dilators of the bucco-labial region. It was while pursuing the course of the latter that we came to the cervical sympathetic. We knew already that they did not belong to the superior maxillary, which Jolyet and Laffont called, wrongly, a typical dilator, that they did not belong even to the nervous system of vital relations, since they were traced to the ganglion of Vieussens, and must be found in the sympathetic of the region of the neck.*

Regarding the sympathetic, Bichât contended that the passions were located in the organs of organic life, and R. M. Bucke, of London, Ontario, asserts that the moral nature of man is seated in the sympathetic system. The close relation between emotional changes and functional derangements of the various viscera led to this idea. "There is nothing new under the sun." Aristotle taught that man had three minds, one of which was seated in the abdomen. The notion that the heart presides over feelings is deeply rooted in the Christian mind, and probably refers to the sympathetic, since nerves alone are capable of sensation. Mr. Rabbinowicz translates thus an article of the *Talmud* of Babylon: "The kidneys give advices, the heart understands, * * the liver is the seat of anger, the gall-bladder throws bile on the latter and calms it, the spleen is the seat of laughter," etc.† It is a fact that diseases of the heart are often simultaneous with ex-

* Revue Médicale, 30 Oct., 1880.

† L'Union Médicale, Oct. 28, 188[illegible].

alted sentiments, that a fit of passion reacts strongly on the circulation, etc. It is also believed by some, that the faculties of the brain are perfected at the expense of a fineness of sentiments. Persons noted for the soundness of their judgment are generally well known for their rudeness, lack of sympathy and of imagination. It would be worth while to examine the sympathetic at autopsies, on the insane, especially the melancholy, and compare the relative development of the same set of nerves in individuals of different temperaments.

M. Foster, in his Text Book of Physiology, third edition, p. 183, says : * * "Division of the cervical sympathetic on one side of the neck causes a dilatation of the minute arteries of the head on the same side, shown by an increased supply of blood to the parts." Then it may be questioned whether the *irritation* (*excitation*) of the French experimenters did not simply incapacitate the nerves, as after section. P. 440, ib, " * * * the numerous phenomena of disease, joined to the facts mentioned above, turn the balance of evidence in favor of the view that some more or less direct influence of the nervous system on metabolic actions, and so on nutrition, will be established by future inquiries."

ACTION OF CARBOLIC ACID UPON CILIATED CELLS AND WHITE BLOOD CELLS. (*Am. Jour. of Med. Sciences*, Jan., 1881.)

Since as far as statistical data are reliable, the results of operations, except in certain classes of cases (ovariotomy, etc.,) are equally good in the hands of those surgeons who use carbolic acid as a dressing agent only, as in the hands of those who adhere strictly to the Listerian doctrines and details, it is a matter of interest to know just what may be the effect of carbolic acid, in varying degrees of strength, upon living tissue. Its beneficial effect may be largely due to its direct action upon the tissues of the healing wound. For experimentation, ciliated and white blood cells were chosen by Dr. T. M. Prudden, since in these the effect of carbolic acid upon the vitality of cells could be most easily seen. He used solutions of varying strength, from 1-20 to 1-3200.

The most extended series of experiments was made upon the tissues of the frog; and less extended ones upon the blood of the rabbit and man. The result of the experiments shows that "carbolic acid in solutions of 1-100 and over, causes either immediately, or in a short time, cessation of vibration in living ciliated cells, with a rapid, characteristic disintegration of their protoplasm and death of the cell." With solutions of 1-50, the ciliary movement ceases in a few seconds, the movements becoming gradually slower and slower. If the movement of the ciliæ is languid, it is temporarily quickened when the acid is first brought in contact with the cell. As the ciliæ cease their motion, degenerative changes occur in the protoplasm of the cells. It is resolved into two kinds of material—one clear, transparent and feebly refractive; the other strongly refractive, appearing as shining globules. "The nucleus shrinks, not infrequently exhibiting, as it does so, distinct movements in the intra-nuclear network; and especially noteworthy in solutions of this strength is the development in a large number of the cells, within a few minutes, of a wedge-shaped, more or less refractive, sharply outlined network, extending from the ciliary border to the nucleus, within which are sometimes seen delicate parallel lines, perpendicular to the free border of the cell. This peculiar network, lying invariably between the ciliary border and the nucleus, and never extending below the latter, would suggest a difference in the structure of the protoplasm in this part of the cell which is not without significance in view of the present uncertainty of our knowledge concerning the relations between the ciliæ and the nucleus and cell body. The shining globules tend to coalesce and gather outside the cell body within the first forty-eight hours."

"In very diluted solutions (1-400 to 1-3200), carbolic acid may cause, if long continued, slight degenerative changes in the protoplasm and the death of the cell; but its most noteworthy action is its inhibitory effect upon ciliary movement. This may be retarded by it, or even entirely checked, without necessarily determining the death of the cell, for on its replacement by an indifferent fluid, the movement may be perfectly re-established and continue indefinitely." These phenomena seem equally true of cells observed *in situ* and of those scraped from their place and teased apart.

The white blood cells were examined, not only when removed from the vessels, but also in the vessels of the tongue, mesentery and bladder of the frog. If the white corpuscles, while being examined in the glass slide, be irrigated with a 1-100 solution, amœboid movement ceases almost instantly, and very soon the protoplasm breaks up into larger and smaller strongly refractive granules or globules, and a finely translucent substance which retains the form of the shrunken cell. The nucleus is more or less jagged in its outline. With weaker solutions the same phenomena are to be observed, but they are developed much more slowly.

If those which have been treated with weak solutions, and whose movements have been stopped, are washed clean with a neutral solution, the movements will usually return.

If a freshly exposed bladder be irrigated with a solution 1-50 to 1-100, it soon becomes cloudy, and stasis will occur almost immediately in the capillaries, gradually extending to the smaller and larger veins and arteries. The red blood cells lose their symmetrical form, becoming permanently cut and curved and occasionally swollen. Sometimes they will cling closely to the sides of the vessel. To these others are joined in little heaps, thus partly blocking up the tubes, or, instead of being thus piled up, the red cells may lie along the wall, side by side, in a single or double layer, thus making a cylindrical investment around the blood current, which still flows, but with impaired velocity. Gradually, however, more and more accumulation takes place, until, finally, the current is stopped. Thus thrombi and emboli are formed partly, as is evident by changes in the red blood cells. The white cells, under these circumstances, do not collect in the peripheral zone of the current, but are mingled indiscriminately with the red ones. They invariably retain their round form. This condition, which has been called "globular stasis," does not undergo resolution, if far advanced.

With solutions of 1-800 to 1-3200 there seems to be no tendency to stasis or thrombosis. If a solution of 1-1600 be used, there is a slight dilatation of both arteries and veins, which seems permanent as long as the part is exposed to the carbolated solution. No change in the capillaries is observed. The current is also at first slightly accelerated, but then becomes slower than normal, and so continues. At first the normal peripheral and axial currents are observed. A greater or less number of white blood cells may be seen rolling or gliding along the walls and even adherent to them. Soon, however, the peripheral zone becomes less and less distinct, and the axial becomes broader. The white cells, which were at first attached to the walls, are loosened, and are carried off by the current. Those which drag along the periphery are mostly globular, and exhibit but slightly the well known appearance of stickiness. This condition lasts as long as the carbolated solution is applied, but the normal phenomena will return if the carbolic acid be washed off with an indifferent fluid. No structural changes could be seen in the white blood cells which had been rendered quiescent in the vessels by solutions as diluted as 1-1600.

It is, therefore, evident that even weak solutions of carbolic acid have the power of checking the emigration of the blood cells; and thus a "part of its favorable action in restraining suppuration may be accounted for, at least in so far as pus is the result of the emigration of the blood cells."

